# Supplementary material for: Pyruvate Dehydrogenase Kinase 1 inhibition mediated oxidative phosphorylation enhancement in cartilage promotes osteoarthritis progression
Source: BMC Musculoskelet Disord. 2023 Jul 20;24:597. doi: 10.1186/s12891-023-06585-6 (PMC10357736; doi:10.1186/s12891-023-06585-6)
Supplement: Supplementary file 1 — Additional file 1. [file 12891_2023_6585_MOESM1_ESM.zip › BP.Gsea.1653623667859/GOBP_RNA_EXPORT_FROM_NUCLEUS.html]

Details for gene set GOBP\_RNA\_EXPORT\_FROM\_NUCLEUS[GSEA]

|  || Dataset | 55457\_collapsed\_to\_symbols.cls.cls #OA\_versus\_normal.cls.cls #OA\_versus\_normal\_repos |
| Phenotype | cls.cls#OA\_versus\_normal\_repos |
| Upregulated in class | normal |
| GeneSet | GOBP\_RNA\_EXPORT\_FROM\_NUCLEUS |
| Enrichment Score (ES) | -0.4664886 |
| Normalized Enrichment Score (NES) | -1.9228727 |
| Nominal p-value | 0.0 |
| FDR q-value | 0.050103035 |
| FWER p-Value | 0.611 |
Table: GSEA Results Summary

  

Fig 1: Enrichment plot: GOBP\_RNA\_EXPORT\_FROM\_NUCLEUS      
 Profile of the Running ES Score & Positions of GeneSet Members on the Rank Ordered List

  

| SYMBOL | TITLE | RANK IN GENE LIST | RANK METRIC SCORE | RUNNING ES | CORE ENRICHMENT || 1 | HHEX | hematopoietically expressed homeobox [Source:HGNC Symbol;Acc:HGNC:4901] | 270 | 0.774 | 0.0116 | No |
| 2 | PCID2 | PCI domain containing 2 [Source:HGNC Symbol;Acc:HGNC:25653] | 1376 | 0.447 | -0.0557 | No |
| 3 | NUP133 | nucleoporin 133 [Source:HGNC Symbol;Acc:HGNC:18016] | 1951 | 0.367 | -0.0850 | No |
| 4 | THOC7 | THO complex 7 [Source:HGNC Symbol;Acc:HGNC:29874] | 2170 | 0.341 | -0.0876 | No |
| 5 | POM121L2 | POM121 transmembrane nucleoporin like 2 [Source:HGNC Symbol;Acc:HGNC:13973] | 2841 | 0.266 | -0.1286 | No |
| 6 | POLDIP3 | DNA polymerase delta interacting protein 3 [Source:HGNC Symbol;Acc:HGNC:23782] | 2869 | 0.264 | -0.1196 | No |
| 7 | NUP93 | nucleoporin 93 [Source:HGNC Symbol;Acc:HGNC:28958] | 3194 | 0.231 | -0.1351 | No |
| 8 | ENY2 | ENY2 transcription and export complex 2 subunit [Source:HGNC Symbol;Acc:HGNC:24449] | 3561 | 0.197 | -0.1553 | No |
| 9 | KHDRBS1 | "KH RNA binding domain containing, signal transduction associated 1 [Source:HGNC Symbol;Acc:HGNC:18116]" | 3748 | 0.182 | -0.1622 | No |
| 10 | NUP155 | nucleoporin 155 [Source:HGNC Symbol;Acc:HGNC:8063] | 3824 | 0.174 | -0.1607 | No |
| 11 | UPF1 | UPF1 RNA helicase and ATPase [Source:HGNC Symbol;Acc:HGNC:9962] | 4434 | 0.118 | -0.2031 | No |
| 12 | DDX19B | DEAD-box helicase 19B [Source:HGNC Symbol;Acc:HGNC:2742] | 4500 | 0.113 | -0.2035 | No |
| 13 | NUP85 | nucleoporin 85 [Source:HGNC Symbol;Acc:HGNC:8734] | 4630 | 0.101 | -0.2092 | No |
| 14 | RAE1 | ribonucleic acid export 1 [Source:HGNC Symbol;Acc:HGNC:9828] | 4697 | 0.096 | -0.2103 | No |
| 15 | NRDE2 | "NRDE-2, necessary for RNA interference, domain containing [Source:HGNC Symbol;Acc:HGNC:20186]" | 5019 | 0.071 | -0.2323 | No |
| 16 | NXF3 | nuclear RNA export factor 3 [Source:HGNC Symbol;Acc:HGNC:8073] | 5648 | 0.020 | -0.2804 | No |
| 17 | TPR | "translocated promoter region, nuclear basket protein [Source:HGNC Symbol;Acc:HGNC:12017]" | 5919 | 0.001 | -0.3014 | No |
| 18 | NUP88 | nucleoporin 88 [Source:HGNC Symbol;Acc:HGNC:8067] | 5976 | -0.003 | -0.3057 | No |
| 19 | GLE1 | GLE1 RNA export mediator [Source:HGNC Symbol;Acc:HGNC:4315] | 6058 | -0.009 | -0.3116 | No |
| 20 | SMG6 | SMG6 nonsense mediated mRNA decay factor [Source:HGNC Symbol;Acc:HGNC:17809] | 6175 | -0.019 | -0.3199 | No |
| 21 | NUP62 | nucleoporin 62 [Source:HGNC Symbol;Acc:HGNC:8066] | 6800 | -0.063 | -0.3658 | No |
| 22 | RBM15B | RNA binding motif protein 15B [Source:HGNC Symbol;Acc:HGNC:24303] | 6867 | -0.069 | -0.3681 | No |
| 23 | SETD2 | "SET domain containing 2, histone lysine methyltransferase [Source:HGNC Symbol;Acc:HGNC:18420]" | 6926 | -0.074 | -0.3695 | No |
| 24 | CHTOP | chromatin target of PRMT1 [Source:HGNC Symbol;Acc:HGNC:24511] | 7184 | -0.095 | -0.3855 | No |
| 25 | NUP62CL | nucleoporin 62 C-terminal like [Source:HGNC Symbol;Acc:HGNC:25960] | 7601 | -0.127 | -0.4126 | No |
| 26 | POLR2D | RNA polymerase II subunit D [Source:HGNC Symbol;Acc:HGNC:9191] | 7726 | -0.136 | -0.4165 | No |
| 27 | NPAP1 | nuclear pore associated protein 1 [Source:HGNC Symbol;Acc:HGNC:1190] | 7737 | -0.136 | -0.4116 | No |
| 28 | CPSF6 | cleavage and polyadenylation specific factor 6 [Source:HGNC Symbol;Acc:HGNC:13871] | 7885 | -0.147 | -0.4168 | No |
| 29 | MCM3AP | minichromosome maintenance complex component 3 associated protein [Source:HGNC Symbol;Acc:HGNC:6946] | 8077 | -0.161 | -0.4249 | No |
| 30 | NOL6 | nucleolar protein 6 [Source:HGNC Symbol;Acc:HGNC:19910] | 8081 | -0.161 | -0.4184 | No |
| 31 | NUP214 | nucleoporin 214 [Source:HGNC Symbol;Acc:HGNC:8064] | 8301 | -0.178 | -0.4279 | No |
| 32 | NCBP2 | nuclear cap binding protein subunit 2 [Source:HGNC Symbol;Acc:HGNC:7659] | 8478 | -0.192 | -0.4335 | No |
| 33 | THOC5 | THO complex 5 [Source:HGNC Symbol;Acc:HGNC:19074] | 8549 | -0.198 | -0.4307 | No |
| 34 | HNRNPA2B1 | heterogeneous nuclear ribonucleoprotein A2/B1 [Source:HGNC Symbol;Acc:HGNC:5033] | 8576 | -0.200 | -0.4243 | No |
| 35 | SUPT6H | "SPT6 homolog, histone chaperone and transcription elongation factor [Source:HGNC Symbol;Acc:HGNC:11470]" | 8752 | -0.213 | -0.4290 | No |
| 36 | DDX25 | DEAD-box helicase 25 [Source:HGNC Symbol;Acc:HGNC:18698] | 8815 | -0.218 | -0.4246 | No |
| 37 | NCBP1 | nuclear cap binding protein subunit 1 [Source:HGNC Symbol;Acc:HGNC:7658] | 9149 | -0.244 | -0.4403 | No |
| 38 | NUP107 | nucleoporin 107 [Source:HGNC Symbol;Acc:HGNC:29914] | 9274 | -0.253 | -0.4393 | No |
| 39 | RAN | "RAN, member RAS oncogene family [Source:HGNC Symbol;Acc:HGNC:9846]" | 9478 | -0.272 | -0.4436 | No |
| 40 | NXF2 | nuclear RNA export factor 2 [Source:HGNC Symbol;Acc:HGNC:8072] | 9577 | -0.281 | -0.4394 | No |
| 41 | XPOT | exportin for tRNA [Source:HGNC Symbol;Acc:HGNC:12826] | 9597 | -0.283 | -0.4290 | No |
| 42 | NXT1 | nuclear transport factor 2 like export factor 1 [Source:HGNC Symbol;Acc:HGNC:15913] | 9983 | -0.319 | -0.4455 | No |
| 43 | SMG5 | SMG5 nonsense mediated mRNA decay factor [Source:HGNC Symbol;Acc:HGNC:24644] | 10190 | -0.340 | -0.4473 | No |
| 44 | SRSF3 | serine and arginine rich splicing factor 3 [Source:HGNC Symbol;Acc:HGNC:10785] | 10280 | -0.349 | -0.4395 | No |
| 45 | DHX9 | DExH-box helicase 9 [Source:HGNC Symbol;Acc:HGNC:2750] | 10627 | -0.386 | -0.4502 | Yes |
| 46 | YTHDC1 | YTH domain containing 1 [Source:HGNC Symbol;Acc:HGNC:30626] | 10687 | -0.391 | -0.4384 | Yes |
| 47 | SMG7 | SMG7 nonsense mediated mRNA decay factor [Source:HGNC Symbol;Acc:HGNC:16792] | 10877 | -0.414 | -0.4357 | Yes |
| 48 | NUP160 | nucleoporin 160 [Source:HGNC Symbol;Acc:HGNC:18017] | 11100 | -0.444 | -0.4343 | Yes |
| 49 | NUP153 | nucleoporin 153 [Source:HGNC Symbol;Acc:HGNC:8062] | 11307 | -0.472 | -0.4305 | Yes |
| 50 | DDX39A | DExD-box helicase 39A [Source:HGNC Symbol;Acc:HGNC:17821] | 11359 | -0.480 | -0.4142 | Yes |
| 51 | NCBP3 | nuclear cap binding subunit 3 [Source:HGNC Symbol;Acc:HGNC:24612] | 11502 | -0.501 | -0.4041 | Yes |
| 52 | TSC1 | TSC complex subunit 1 [Source:HGNC Symbol;Acc:HGNC:12362] | 11575 | -0.511 | -0.3882 | Yes |
| 53 | ZC3H11A | zinc finger CCCH-type containing 11A [Source:HGNC Symbol;Acc:HGNC:29093] | 11780 | -0.546 | -0.3811 | Yes |
| 54 | DDX19A | DEAD-box helicase 19A [Source:HGNC Symbol;Acc:HGNC:25628] | 11821 | -0.553 | -0.3609 | Yes |
| 55 | NUP188 | nucleoporin 188 [Source:HGNC Symbol;Acc:HGNC:17859] | 11877 | -0.567 | -0.3413 | Yes |
| 56 | THOC6 | THO complex 6 [Source:HGNC Symbol;Acc:HGNC:28369] | 11918 | -0.576 | -0.3202 | Yes |
| 57 | SSB | small RNA binding exonuclease protection factor La [Source:HGNC Symbol;Acc:HGNC:11316] | 11955 | -0.584 | -0.2984 | Yes |
| 58 | AGFG1 | ArfGAP with FG repeats 1 [Source:HGNC Symbol;Acc:HGNC:5175] | 12035 | -0.602 | -0.2792 | Yes |
| 59 | POM121 | POM121 transmembrane nucleoporin [Source:HGNC Symbol;Acc:HGNC:19702] | 12146 | -0.631 | -0.2612 | Yes |
| 60 | UPF2 | UPF2 regulator of nonsense mediated mRNA decay [Source:HGNC Symbol;Acc:HGNC:17854] | 12150 | -0.631 | -0.2348 | Yes |
| 61 | SMG1 | SMG1 nonsense mediated mRNA decay associated PI3K related kinase [Source:HGNC Symbol;Acc:HGNC:30045] | 12268 | -0.668 | -0.2158 | Yes |
| 62 | HNRNPA1 | heterogeneous nuclear ribonucleoprotein A1 [Source:HGNC Symbol;Acc:HGNC:5031] | 12356 | -0.693 | -0.1934 | Yes |
| 63 | NXF1 | nuclear RNA export factor 1 [Source:HGNC Symbol;Acc:HGNC:8071] | 12410 | -0.710 | -0.1676 | Yes |
| 64 | PABPN1 | poly(A) binding protein nuclear 1 [Source:HGNC Symbol;Acc:HGNC:8565] | 12461 | -0.730 | -0.1407 | Yes |
| 65 | THOC1 | THO complex 1 [Source:HGNC Symbol;Acc:HGNC:19070] | 12503 | -0.749 | -0.1124 | Yes |
| 66 | NPM1 | nucleophosmin 1 [Source:HGNC Symbol;Acc:HGNC:7910] | 12530 | -0.762 | -0.0823 | Yes |
| 67 | AKAP8L | A-kinase anchoring protein 8 like [Source:HGNC Symbol;Acc:HGNC:29857] | 12665 | -0.836 | -0.0575 | Yes |
| 68 | THOC2 | THO complex 2 [Source:HGNC Symbol;Acc:HGNC:19073] | 12691 | -0.855 | -0.0234 | Yes |
| 69 | DDX39B | DExD-box helicase 39B [Source:HGNC Symbol;Acc:HGNC:13917] | 12783 | -0.941 | 0.0091 | Yes |
Table: GSEA details [plain text format]

  

Fig 2: GOBP\_RNA\_EXPORT\_FROM\_NUCLEUS      
 Blue-Pink O' Gram in the Space of the Analyzed GeneSet

  

Fig 3: GOBP\_RNA\_EXPORT\_FROM\_NUCLEUS: Random ES distribution      
 Gene set null distribution of ES for **GOBP\_RNA\_EXPORT\_FROM\_NUCLEUS**
